# A single mild closed-head injury disrupts synaptic strength and promotes hippocampal hyperexcitability in mice

**DOI:** 10.1093/braincomms/fcag268

**Published:** 2026-07-12

**Authors:** Jonathan C Vincent, Matthew J Lanning, Kai Saito, Cate D Cox, Leke Bytyqi, Savannah M Shepard, Blake K Byer, Margaret R Hawkins, Teresa Macheda, Kelly N Roberts, Heather M Hash, Kristen A McLaurin, Josh M Morganti, Christopher M Norris, Adam D Bachstetter

**Affiliations:** Department of Neuroscience, University of Kentucky, Lexington, KY 40536-0509, USA; Spinal Cord and Brain Injury Research Center, University of Kentucky, Lexington, KY 40536-0509, USA; Sanders-Brown Center on Aging, University of Kentucky, Lexington, KY 40536-0509, USA; Spinal Cord and Brain Injury Research Center, University of Kentucky, Lexington, KY 40536-0509, USA; Sanders-Brown Center on Aging, University of Kentucky, Lexington, KY 40536-0509, USA; Spinal Cord and Brain Injury Research Center, University of Kentucky, Lexington, KY 40536-0509, USA; Spinal Cord and Brain Injury Research Center, University of Kentucky, Lexington, KY 40536-0509, USA; Spinal Cord and Brain Injury Research Center, University of Kentucky, Lexington, KY 40536-0509, USA; Spinal Cord and Brain Injury Research Center, University of Kentucky, Lexington, KY 40536-0509, USA; Spinal Cord and Brain Injury Research Center, University of Kentucky, Lexington, KY 40536-0509, USA; Spinal Cord and Brain Injury Research Center, University of Kentucky, Lexington, KY 40536-0509, USA; Spinal Cord and Brain Injury Research Center, University of Kentucky, Lexington, KY 40536-0509, USA; Spinal Cord and Brain Injury Research Center, University of Kentucky, Lexington, KY 40536-0509, USA; Department of Pharmaceutical Sciences, University of Kentucky, Lexington, KY 40536-0509, USA; Department of Neuroscience, University of Kentucky, Lexington, KY 40536-0509, USA; Spinal Cord and Brain Injury Research Center, University of Kentucky, Lexington, KY 40536-0509, USA; Sanders-Brown Center on Aging, University of Kentucky, Lexington, KY 40536-0509, USA; Department of Neuroscience, University of Kentucky, Lexington, KY 40536-0509, USA; Sanders-Brown Center on Aging, University of Kentucky, Lexington, KY 40536-0509, USA; Department of Pharmacology and Nutritional Sciences, University of Kentucky, Lexington, KY 40536-0509, USA; Department of Neuroscience, University of Kentucky, Lexington, KY 40536-0509, USA; Spinal Cord and Brain Injury Research Center, University of Kentucky, Lexington, KY 40536-0509, USA; Sanders-Brown Center on Aging, University of Kentucky, Lexington, KY 40536-0509, USA

**Keywords:** diffuse brain injury, sex differences, transcriptomics, field potentials, spine morphology

## Abstract

Traumatic brain injury can result in persistent cognitive, behavioural, and emotional deficits, with the hippocampus among the most vulnerable circuits after injury. However, how diffuse injury differentially alters hippocampal subregions across time remains incompletely defined. Here, we used a mouse closed-head injury model to characterize early transcriptomics, subacute-to-chronic electrophysiology, dendritic spine morphology, and delayed immunoreactivity for glial fibrillary acidic protein (GFAP), ionized calcium-binding adapter molecule 1 (IBA1), and the pan-leukocyte marker CD45. Bulk RNA sequencing at 9, 24, and 72 h post-injury revealed induction of immediate early genes and neuronal excitability transcripts at 9 h alongside inflammatory pathways. These neuronal signatures diminished by 24–72 h while immune-associated programs persisted. Ex vivo field recordings in CA1 and dentate gyrus at 1, 3, and 6 weeks post-injury revealed reductions in synaptic strength in both regions at 1 week. Dentate gyrus deficits persisted at 3 weeks but recovered by 6 weeks, whereas CA1 showed depression at 1 and 6 weeks with relative sparing at 3 weeks. Fibre volley recruitment was preserved across regions and timepoints, arguing against gross presynaptic loss. Population spike thresholds were reduced in both regions, indicating increased neuronal excitability that persisted in CA1 but partially recovered in dentate gyrus. DiOlistic labelling and spine reconstruction revealed stable total spine density, but spine class composition showed sex-dependent injury effects in CA1 with altered mushroom and stubby proportions in males. Immunohistochemistry across 1–8 weeks post-injury revealed cortical gliosis but no injury-related changes in hippocampal GFAP or IBA1, while CD45 immunoreactivity increased in a delayed, sex-dependent manner within hippocampus. Together, these findings show that a single closed-head injury produces sustained hippocampal circuit dysfunction characterized by reduced synaptic strength and increased neuronal excitability, with region-dependent recovery dynamics, preserved presynaptic recruitment, and delayed hippocampal CD45 increases that do not parallel local glial activation.

## Teaser

Vincent *et al.* report that a mild diffuse brain injury causes lasting hippocampal synaptic dysfunction and hyperexcitability. These deficits persist after early transcriptional changes resolve and are accompanied by sex-dependent spine remodelling and delayed neuroinflammatory activation, occurring independently of the overt glial response typically associated with brain trauma.

## Graphical Abstract

**Graphical Abstract:**
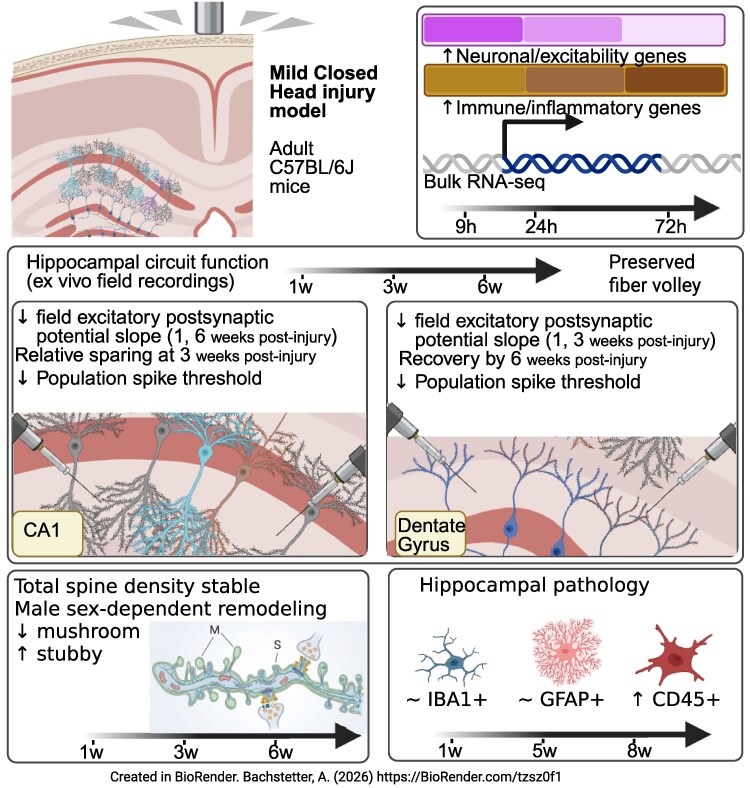

## Introduction

Traumatic brain injury (TBI) can cause long-term neurological disability, including persistent cognitive, behavioural, and emotional deficits.^1-4^ TBI rapidly engages inflammatory cascades and activity-dependent neuronal transcriptional responses, and these early molecular changes may shape later circuit dysfunction.^5-8^ With early responses to excitotoxicity, energy failure, mechanical stretch, oedema, and inflammation, neurons undergo persistent alterations in synaptic function (e.g. synapse loss and impaired synaptic plasticity) resulting in improper circuit remodelling, which likely contribute to the long-term sequelae of TBI, including increased neuronal excitability and an elevated risk of post-traumatic seizures and epilepsy.^9-15^ Injury forces and their severity shape distinct primary insults to the brain and, in turn, determine how synapses are affected and how neurons attempt to compensate and remodel in response to these injuries.^16,17^ However, despite this heterogeneity, few studies have examined mild diffuse closed-head injury (CHI), the most common form of TBI experienced by individuals.

The hippocampus, despite not often being directly subjected to mechanical impact in many TBI models, is particularly vulnerable to injury-related forces.^18,19^ The hippocampus is composed of subregions with distinct cytoarchitecture and circuit functions that may confer differential vulnerability and recovery after injury.^20-23^ For instance, CA1 pyramidal neurons receive Schaffer collateral input from CA3 and provide major hippocampal output to cortical targets, whereas dentate gyrus (DG) granule cells gate entorhinal inputs and regulate downstream hippocampal recruitment through sparse coding.^24-27^ Studies have consistently shown that CA1 is particularly susceptible to excitotoxic and metabolic stress, displaying pronounced synaptic depression and dendritic retraction after trauma.^14,28-33^ In contrast, the DG often demonstrates transient suppression or even compensatory hyperexcitability, reflecting potential resilience or neurogenic plasticity.^26,34-37^ At the synaptic level, dendritic spines are the principal postsynaptic sites of excitatory transmission and undergo dynamic remodelling after TBI.^38-40^ Many studies report reduced mushroom-type spines and increased, less mature spine morphologies, consistent with altered synaptic stability and efficacy.^38,41,42^ Prior studies have demonstrated alterations in hippocampal synaptic physiology following TBI, including impaired long-term potentiation,^14,15,32^ altered excitatory drive,^26,28,30,43,44^ and increased network excitability,^45-49^ often with a focus on individual subregions or limited timepoints.^24,26,27^ These findings highlight the sensitivity of hippocampal circuits to injury but are typically restricted to single regions or endpoints. As a result, the relationship between synaptic physiology, structural remodelling, and region-specific recovery trajectories following mild diffuse injury remains incompletely defined, particularly across hippocampal subregions and with consideration of sex as a biological variable.

The CHI model used here reproduces diffuse biomechanical forces relevant to brain injury without craniotomy, preserving the skull and meninges while producing robust functional deficits.^50^ Because early injury-induced transcriptional responses may shape later circuit dysfunction, we used bulk RNA sequencing to define the acute molecular response to CHI and then examined hippocampal electrophysiology at later subacute-to-chronic timepoints to determine whether persistent synaptic abnormalities emerge after these early molecular changes. Early timepoints (9, 24, and 72 h) were selected to capture the acute transcriptional response to injury, including activity-dependent and inflammatory gene expression changes. Later timepoints (1, 3, and 6 weeks post-injury) were chosen to assess subacute-to-chronic alterations in hippocampal circuit function during periods when behavioural deficits are present. At these later timepoints, we quantified synaptic strength, presynaptic recruitment, postsynaptic responses, and population spike threshold in CA1 and DG. Together, these timepoints were selected to capture distinct phases of the injury response. To assess structural correlates, we performed DiOlistic labelling and three-dimensional spine reconstruction in a subset of animals to quantify spine morphology composition (percent mushroom and percent stubby) and tested structure-function relationships using correlation analyses. Finally, we evaluated hippocampal and cortical immunoreactivity for GFAP, IBA1, and CD45 at 1, 5, and 8 weeks post-injury to determine whether signs of gliosis and neuroinflammation were present within hippocampal subregions. Together, this multi-level approach was designed to (i) define the early molecular response to CHI, (ii) determine whether CA1 and DG exhibit distinct temporal profiles of synaptic dysfunction, (iii) assess whether spine morphology composition and its relationship to physiology differs by sex, and (iv) evaluate whether hippocampal neuroimmune marker expression parallels or diverges from robust cortical pathology after CHI.

## Materials and methods

### Animals, ethics, and study design

All procedures were approved by the University of Kentucky Institutional Animal Care and Use Committee and adhere to the ARRIVE guidelines 2.0.^51^ Adult male and female C57BL/6J mice were housed on a 12-hour light/dark cycle with ad libitum food and water. Mice were allocated to independent cohorts for bulk RNA sequencing, electrophysiology/spine analysis, or histology, with cohort-specific sample sizes and timepoints described below. Animals were randomly assigned to experimental groups within each cohort. Data collection and quantification were performed blinded to group until completion of initial analyses.

### Closed-head injury and sham surgery

CHI was performed as previously described.^50^ For surgical procedures, animals in the sham and CHI groups were anesthetized with 2.5% isoflurane, their heads were shaved, protective eye ointment was applied, and the scalp was disinfected with a betadine solution. Throughout the procedure, core body temperature was maintained at 37°C using a controlled heating pad. Mice were secured in a stereotaxic frame (Stoelting Co., Wood Dale, USA) using non-traumatic ear bars, with anaesthesia maintained at 2.5% isoflurane via a non-rebreathing nasal cone. To displace the impact force from the ear bars, a 1-mL latex pipette bulb filled with water was positioned directly under the mouse’s head. A midline scalp incision was made to expose the skull. For the CHI group, a single, controlled impact was delivered to the intact skull using a 5.0 mm steel-tip impactor at coordinates ML = 0.0 mm and AP = −1.6 mm (relative to bregma), with an impact velocity of 5.0 ± 0.2 m/s and an impact depth of 1.0 mm. Mice in the sham group underwent all identical procedures, including anaesthesia and scalp incision, but received no impact. Righting reflex time was measured immediately after injury as the time required for the animal to return to a prone position following removal from anaesthesia. No skull fractures were observed in this study, and no animals were excluded on this basis.

### RNA sequencing

A total of 61 adult C57BL/6J mice (31 male, 30 female; age 5.12 ± 0.76 months; weight 27.24 ± 4.74 g; mean ± SD) were used for bulk RNA sequencing. Mice underwent CHI or sham surgery as described above and were euthanized at 9 , 24 , or 72 h post-injury. All sham and CHI procedures were done in the morning, 08:00–10:00. Accordingly, tissue collection at 9, 24, and 72 h post-procedure occurred at different times of day, which may contribute to time-of-day-dependent variation in activity-regulated and synaptic gene expression. CHI group sizes were 7 females and 8 males (9 h), 8 females and 6 males (24 h), and 6 females and 8 males (72 h). Sham group sizes were 3 females and 3 males at each timepoint. Mice were perfused with ice-cold 1× PBS, and one hemisphere was snap-frozen for RNA extraction. Total RNA was isolated using the QIAGEN RNeasy Mini Kit and shipped to Novogene (Sacramento, CA) for library preparation and sequencing. All samples met quality thresholds (RIN range 8.7–9.7). Poly(A)-selected mRNA libraries were prepared using Novogene’s standard workflow and sequenced on an Illumina NovaSeq X Plus platform to generate paired-end reads. Raw sequencing data were processed using the nf-core/rnaseq pipeline (v3.21.0).^52^ After trimming, samples yielded ∼30–60 million reads each. Sequencing quality was assessed using MultiQC; all samples met quality thresholds with no technical outliers. Trimmed reads were aligned to the GRCm39 mouse reference genome using STAR, achieving 85–95% total mapping and 75–85% unique mapping. Unmapped reads (too short or other categories) represented < 10% of read pairs. Transcript quantification was performed using Salmon.^53^ Gene counts and lengths were imported into DESeq2 (v1.46.0) using the DESeqDataSetFromTximport function to generate gene length offsets.^54,55^ Prior to differential expression analysis, genes were filtered to retain those with ≥10 counts in at least *n* samples, where *n* equals the smallest group size in each comparison. The DESeq2 model included sex and age as covariates.^55^ Differentially expressed genes (DEGs) were defined as those with adjusted *P*-value < 0.05 (Benjamini–Hochberg correction). For downstream pathway analysis, an additional threshold of absolute log2 fold-change > 0.5 was applied to further filter the gene set. Over-representation analysis was performed separately for upregulated and downregulated DEGs using clusterProfiler v4.14.6 (GO Biological Process, KEGG) and ReactomePA v1.50.0 (Reactome).^56-58^ Neuronal-related gene sets (immediate early genes, neuronal excitability genes, regulatory factors, and synaptic function genes) were annotated using curated references. MA plots, volcano plots, Venn diagrams, expression trajectories, and enrichment profiles were generated with ggplot2 and standard plotting functions. MultiQC outputs were used exclusively for sequencing quality control and were not incorporated into downstream statistical modelling.

### Electrophysiology and dendritic spine analysis

A total of 120 adult C57BL/6J mice (55 male, 65 female; age 4.66 ± 0.75 months; weight 27.48 ± 5.24 g; mean ± SD) were used for electrophysiology experiments and a random subset of 33 mice underwent paired dendritic spine analyses. Mice underwent CHI or sham surgery as described above and were studied at 1, 3, or 6 weeks post-injury (wkpi; 1.25 ± 0.44, 3.30 ± 0.39, and 5.99 ± 0.89 wkpi; mean ± SD). Mice assigned to the naïve group were left undisturbed. For CHI, group sizes were 10 females and 8 males (1 wkpi), 10 females and 10 males (3 wkpi), and 12 females and 7 males (6 wkpi). Naïve controls comprised 8 females and 13 males, and sham controls comprised 25 females and 17 males.

#### Hippocampal slice preparation and recovery

Hippocampal slice preparation was performed as described in our earlier work.^14^ At 1, 3, or 6 wkpi, mice were anesthetized under CO_2_ and decapitated. Brains were rapidly extracted, and submerged (within 44.3 ± 7.68 s after decapitation; mean ± SD) in ice-cold, oxygenated (95% O_2_, 5% CO_2_) artificial cerebrospinal fluid (ACSF) slicing solution. The cutting solution contained (in mM): 114 NaCl, 3.0 KCl, 10.0 glucose, 1.25 KH2PO4, 26 NaHCO3, and 2.0 MgSO4 (pH 7.4; ∼300 mOsm). Brains were hemisected and cut coronally into 400 µm slices using a Leica VT1200 S vibratome (Leica Biosystems). The hemisections designated for electrophysiology were transferred to a custom interface holding chamber (Protoscience Solutions) for recovery and incubated in warm (∼32°C), oxygenated ACSF containing 2 mM CaCl_2_. Slices recovered for 2–5 h prior to recording. The other hemisection was reserved for dendritic spine analysis (see *Dendritic spine analysis* below).

#### Field potential recordings in the CA1 and dentate gyrus

For each animal, 1–3 slices per hippocampal region (CA1 and DG) were recorded when available. Recordings from CA1 and DG were obtained from the same animals when possible; however, not all animals yielded usable recordings in both regions. For each endpoint, slice-level measurements were averaged within each animal for each region prior to statistical analysis, and animal-level values were used for statistical comparisons. Slices were perfused in oxygenated ACSF (∼32°C) at a rate of 1–2 mL/min in an RC26 chamber (Warner Instrument). Stimuli were delivered via bipolar stainless-steel electrodes using a constant-current stimulus isolation unit (A365; World Precision Instruments). For CA1, Schaffer collaterals were stimulated in stratum radiatum near the CA3-CA1 border; for DG, perforant path inputs were stimulated near the DG crest. Slices were included only if the field excitatory post-synaptic potential (fEPSP) at 200 µA was ≥0.5 mV. fEPSPs were recorded in CA1 stratum radiatum and DG molecular layer using glass micropipettes (1–6 MΩ) filled with ACSF. Signals were amplified, filtered, and digitized using a Multiclamp 700B and Digidata 1550B (Molecular Devices). Input-output curves were generated using 100 µs pulses at eleven stimulus intensities (25–600 µA) at 0.1 Hz, with 3–5 responses averaged per intensity. Stimulus timing and data acquisition were controlled by Clampex software (v11.2.2.17; Molecular Devices). Fibre volley (FV) amplitude and fEPSP slope were measured offline in LabChart (ADInstruments). fEPSP slope was quantified as the maximum slope of the initial descending phase of the field potential (i.e. the steepest linear portion of the monosynaptic response), measured within a cursor window positioned on the early descending limb prior to population spike onset. Synaptic strength was quantified as the slope of fEPSP versus FV amplitude. Presynaptic excitability was quantified as FV amplitude versus stimulus intensity. Postsynaptic responsiveness was quantified as fEPSP slope versus stimulus intensity. Synaptic efficiency was calculated as fEPSP/FV ratio at the three highest stimulation intensities. Population spike threshold was defined as the fEPSP slope at which a population spike first emerged in the ascending limb of the field potential and served as an index of neuronal excitability.

### Dendritic spine analysis

Dendritic spine analyses were performed on a subset of animals from the electrophysiology cohort (*n* = 33) using paired tissue collected at the time of slice preparation. Coronal hemisections were drop-fixed in 4% PFA for 10–12 min and transferred to PBS until DiOlistic labelling (within 24–48 h). DiOlistic labelling was performed using adapted methods.^59,60^ DiI-coated tungsten particles were prepared and loaded into Tefzel tubing cartridges. Fixed 400 µm slices were labelled using a Helios Gene Gun (Bio-Rad) at ∼90 psi helium. Sections were mounted with ProLong Gold Antifade (Invitrogen) and stored at 4°C until imaging. Confocal imaging was performed on a Nikon A1R HD laser-scanning microscope. Hippocampal subregions were localized at 10×, and well-isolated neurons were imaged at 60× with *z*-stacks collected at 0.10–0.15 µm step size. Three to five dendritic segments per region per animal were imaged under blinded conditions. Dendritic spine analyses were performed on CA1 pyramidal neuron dendritic segments selected based on imaging and reconstruction suitability rather than formal compartment classification. Segments were at least second-order branches, were not directly attached to the soma or main apical dendrite, were largely contained within a single z-plane, and provided at least 40 μm of dendritic length that was not obscured by neighbouring dendrites. Spine density and morphology were quantified using Imaris v9.9.1 (Oxford Instruments). For each segment, a 40 μm dendritic length was traced manually, and spines were manually seeded in three dimensions to reduce false positives. Spines were classified as mushroom (max_width(head) ≥ min_width(neck); 1 μm < length < 3 μm) or stubby (length < 1 μm) using predefined geometric rules validated across >20 representative segments.^61^ Spines not meeting either classification were designated as ‘other.’ This category likely included morphologies such as filopodia- or nubby-like spines based on classic spine classification criteria; however, these morphologies could not be reliably separated into distinct subclasses using the software-based analysis workflow and therefore were not analyzed as independent categories. Spine composition was calculated as percent mushroom and percent stubby relative to the total detected spine population within each segment, with ‘other’ spines retained in the denominator but not analyzed as an independent morphological category.

### Histology and immunohistochemistry

Histopathological analyses were performed using archived brain tissue from an independent cohort of C57BL/6J sham and CHI mice collected at 1, 5, and 8 wkpi (*n* = 74; 39 male, 35 female). This cohort was distinct from the RNA-seq and electrophysiology/spine cohorts and was used as a complementary assessment of delayed hippocampal pathology. Use of archived tissue is also consistent with the 3Rs principle of reduction. Mice were transcardially perfused with 1X PBS followed by 4% PFA. Brains were post-fixed, cryoprotected in 30% sucrose, and sectioned coronally at 40 µm on a freezing microtome. For immunohistochemistry, every tenth section spanning dorsal hippocampus (∼−1.3 to −2.5 mm from bregma) was selected. Free-floating sections were processed using established protocols^62^ with primary antibodies targeting GFAP (rabbit, 1:10 000; Dako Z0334; RRID: AB_10013382), IBA1 (rabbit, 1:10 000; Wako 019-19741; RRID: AB_839504), and CD45 (rat, 1:1000; BioLegend 103102; RRID: AB_312967). Signal was amplified using VECTASTAIN Elite ABC-HRP and developed with DAB (Vector Laboratories). Slides were scanned using a Zeiss AxioScan Z.1, and percent area of positive staining was quantified in HALO (Indica Labs) using the Area Fraction algorithm. Percent area of positive staining was defined as the proportion of threshold-positive pixels relative to the total area of the delineated region of interest. CA1 and DG were delineated using consistent anatomical boundaries across sections. All sections were processed and imaged in batch under identical conditions. Quantification was performed blinded to experimental group using uniform thresholding parameters across all samples, and animals were randomized across staining batches to minimize potential batch effects.

### Statistical analysis

Statistical analyses were performed in R (v4.4.2) or JMP Student Edition 18 (SAS Institute Inc.), and graphs were generated in GraphPad Prism 10 (GraphPad Software LLC) or R. For RNA sequencing, gene-level counts were analyzed in DESeq2, with differential expression tested using Wald statistics for CHI versus sham at each timepoint (9, 24, 72 h) and Benjamini-Hochberg correction (FDR < 0.05). Functional enrichment analyses used Gene Ontology (GO) biological processes, KEGG, and Reactome pathway annotations. For electrophysiology, slice-level measurements were averaged within each animal for each region before statistical testing, and animal-level values were used for inference. Electrophysiology endpoints were analyzed using two-way ANOVA (injury group × sex) within each hippocampal region, with Dunnett's post hoc comparisons against sham. For dendritic spines, spine morphology (percent mushroom, percent stubby) was analyzed by ANOVA within each region with Tukey’s post hoc comparisons, and Pearson correlations were computed between spine morphology and electrophysiology measures separately by region and sex. For immunohistochemistry, percent area fraction was analyzed using mixed-effects ANOVA with animal as a random intercept and fixed effects for injury, timepoint, region, and sex, with Dunnett’s or Tukey’s correction for post hoc comparisons. Data are presented as mean ± SEM unless otherwise indicated. Model assumptions were assessed prior to inference, and statistical significance was set at *α* = 0.05.

## Results

### Closed-head injury induces an early time-dependent transcriptional response

**Figure 1:**
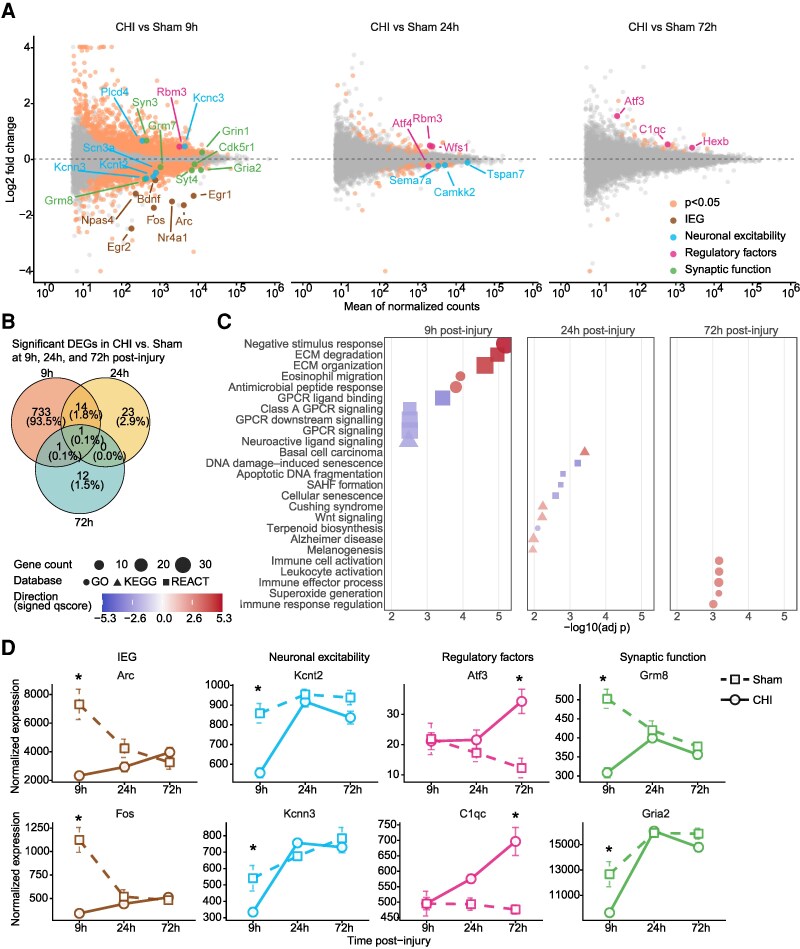
Early transcriptional responses to CHI. (**A**) MA plots showing DEGs at 9, 24, and 72 h after CHI. The 9 h time point exhibited the largest transcriptional response. Each point represents an individual gene. Differential expression was tested in DESeq2 using the Wald test with Benjamini–Hochberg correction. Neuronal-related transcripts highlighted in the plots were selected *a priori* based on their roles in immediate early signalling, neuronal excitability, and synaptic function. (**B**) Venn diagram showing minimal overlap in DEGs across time points, with most changes occurring at 9 h and only 14 DEGs detected at 72 h. (**C**) Pathway enrichment analysis demonstrating early activation of extracellular matrix, GPCR, and neuroactive ligand pathways at 9 h, and enrichment of senescence-, oxidative stress-, and disease-related pathways at later time points. (**D**) Normalized expression trajectories for representative neuronal and injury-associated genes. Immediate early, excitability-related, and synaptic transcripts peaked at 9 h and declined thereafter, whereas Atf3 and the microglial complement gene C1qc remained elevated through 72 h. Each point represents the group mean normalized expression at that timepoint (circles, CHI; squares, Sham); error bars represent the standard error of the mean. Differential expression was tested in DESeq2 using the Wald test with Benjamini–Hochberg correction. * FDR-adjusted *P* < 0.05 (DESeq2, Benjamini–Hochberg). Animals examined per group: 9 h CHI, 7 female/8 male; 24 h CHI, 8 female/6 male; 72 h CHI, 6 female/8 male; Sham, 3 female/3 male at each timepoint.

To define the early molecular responses triggered by CHI and establish a baseline framework for interpreting downstream region-specific synaptic physiology, we first performed bulk RNA sequencing at 9, 24, and 72 h post-injury, time points selected based on prior work identifying peak transcriptional changes after CHI.^63^ Bulk RNA-seq revealed a series of time-specific transcriptional changes with the most changes at 9 h post-injury, and fewer DEGs at 24 and 72 h (Fig. 1A). Because our overarching goal is to understand how CHI alters neuronal and synaptic function, we highlighted a set of neuronal-related genes in the MA plots based on their established roles in immediate early signalling, neuronal excitability, and synaptic transmission. These genes (e.g. *Arc*, *Fos*, *Egr1/2*, *Kcnt2*, *Kcnc3*, *Gria2*, *Grm8*, *Syt4*) were selected *a priori* for their biological relevance to our experimental questions rather than through an unbiased enrichment approach. Their coordinated response illustrates a rapid phase of neuronal activation following CHI. At 9 h, transcripts associated with neuronal activity and excitability were prominently upregulated, including immediate early genes (*Arc, Fos, Egr1/2*), ion channel regulators (*Kcnt2, Kcnc3*), and synaptic function genes (*Gria2, Grm8, Syt4*). This early activity-linked signature was substantially diminished by 24 h and largely resolved by 72 h, indicating a rapid but transient neuronal activation phase. In contrast, genes associated with injury-related regulatory programs and glial engagement showed more prolonged alterations. Factors linked to neuronal stress and axonal injury (*Atf3*) and microglial complement components (*C1qc*) remained elevated through 72 h, consistent with evolving cellular responses after CHI. Across the three time points, the overlap in DEGs was minimal (Fig. 1B). Notably, only 14 genes reached significance at 72 h, and 12 of these were unique to that timepoint. Pathway analyses supported this interpretation. The 9 h dataset was dominated by pathways involved in extracellular matrix remodelling, GPCR and neuroactive ligand signalling, and other processes linked to rapid neuronal activation (Fig. 1C). In contrast, the 24 and 72 h datasets showed enrichment for pathways associated with cellular senescence, apoptosis, oxidative stress responses, and disease-related signatures, including Alzheimer’s disease-linked pathways. To illustrate temporal patterns within representative gene classes, we examined normalized expression for selected transcripts (Fig. 1D). Immediate early genes (*Arc, Fos*), neuronal excitability regulators (*Kcnt2, Kcnc3*), and synaptic genes (*Gria2, Grm8*) reached maximal induction at 9 h and returned toward baseline by 24–72 h. Conversely, injury-associated regulators (*Atf3*) and microglial complement components (*C1qc*) showed persistent or increasing expression at the later timepoints. Sham mice displayed expected circadian fluctuations in activity-dependent transcripts, consistent with established rhythmic regulation of neuronal-related gene expression. Together, these data indicate that CHI elicits a rapid, time-structured transcriptional response within the first 72 h. Early responses include transient neuronal activity-linked programs, whereas later profiles are dominated by immune- and injury-associated pathways.

### Closed-head injury induces lasting alterations in hippocampal synaptic electrophysiology

**Figure 2:**
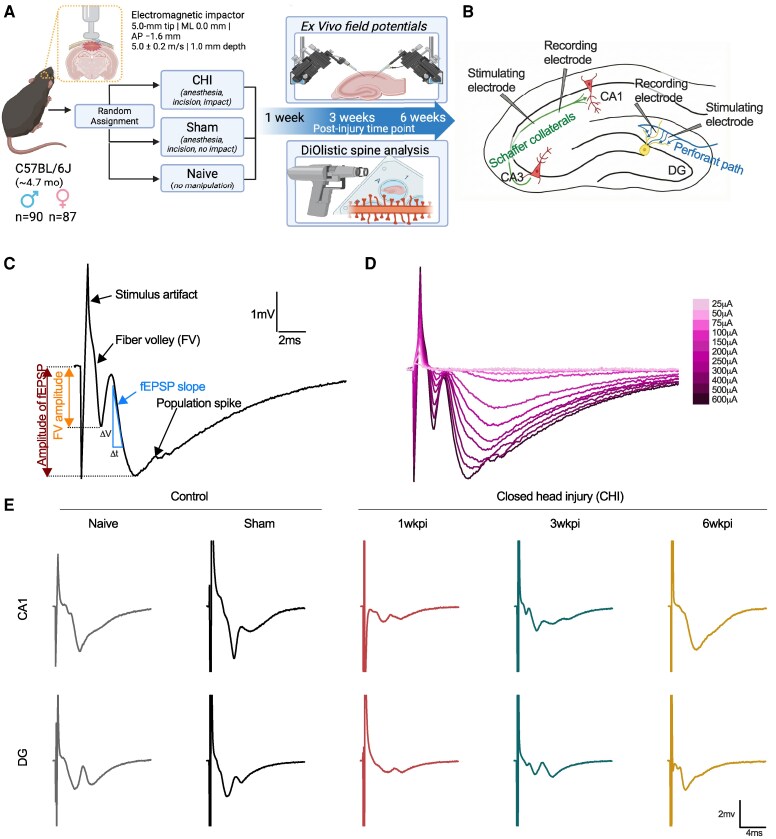
Experimental design and ex vivo assessment of hippocampal synaptic function following CHI. (**A**) Experimental timeline and study design was created in BioRender. Bachstetter, A. (2026) https://BioRender.com/2uufhqc. Male and female C57BL/6 mice were randomly assigned to Naïve (no manipulation), Sham (anaesthesia and scalp incision), or CHI (CHI; stereotaxic electromagnetic impactor) groups. CHI was delivered using a 5.0-mm tip at coordinates ML 0.0 mm and AP −1.6 mm, with an impact velocity of 5.0 ± 0.2 m/s and a depth of 1.0 mm. Animals were evaluated at 1, 3, or 6 weeks post-injury (wkpi). Brains were hemisected, with one hemisphere used for ex vivo hippocampal slice electrophysiology and the contralateral hemisphere used for DiOlistic labelling and dendritic spine analysis. (**B**) Schematic of hippocampal field potential recording configurations. Field excitatory postsynaptic potentials (fEPSPs) were recorded in CA1 stratum radiatum following stimulation of Schaffer collateral fibres originating from CA3, and in the DG (DG) molecular layer following stimulation of the perforant path. (**C**) Annotated representative fEPSP trace illustrating key waveform components, including the stimulus artefact, presynaptic fibre volley (FV), fEPSP slope, fEPSP amplitude, and population spike, which reflects synchronous neuronal firing. (**D**) Representative input–output curve generated from a single hippocampal slice, showing overlaid fEPSP traces averaged from 3–5 sweeps at increasing stimulation intensities (25–600 µA). (**E**) Representative fEPSP traces recorded at the maximal stimulation intensity (600 µA) from CA1 (top row) and DG (bottom row) across experimental groups (Naïve, Sham, 1 wkpi, 3 wkpi, and 6 wkpi). Scale bars in (**C**) apply to panels (**C**) and (**D**); scale bars in (**E**) are shown at right.

The overarching goal of this study was to determine how neuronal activity is altered following a single mild CHI and whether early transcriptional responses, including those associated with synaptic dysfunction and neuronal excitability changes (Fig. 1) and previously observed long-lasting cognitive and behavioural deficits,^8,63^ correspond to persistent alterations in synaptic circuitry. We focused on the hippocampus as a well-defined, highly injury-sensitive circuit that is directly affected by CHI^20-23^ to test circuit-level synaptic changes following a diffuse TBI. Building on our prior work demonstrating behavioural impairments lasting up to 14 weeks after CHI,^63^ with deficits in multiple hippocampal-associated behavioural assays evident through at least 3 weeks post-injury (wkpi), we examined hippocampal synaptic function at timepoints before (1 wkpi), during (3 wkpi), and after (6 wkpi) the period in which these behavioural deficits are observed (Fig. 2). Adult male and female C57BL/6 mice (age: 4.66 ± 0.75 months; weight: 27.48 ± 5.24 g; mean ± SD) were assigned to naïve, sham, or CHI groups and assessed at 1, 3, or 6 weeks post-injury. Injury severity was confirmed by righting reflex time measured immediately after impact. When stratified by sex, righting reflex time was prolonged in CHI animals in both females and males. In females, righting reflex time was 7.66 ± 5.04 s at 1 wkpi (*n* = 11), 13.15 ± 7.03 s at 3 wkpi (*n* = 16), and 12.83 ± 9.65 s at 6 wkpi (*n* = 15), compared with 1.49 ± 0.66 s in sham controls (*n* = 32). In males, righting reflex time was 12.44 ± 8.43 s at 1 wkpi (*n* = 11), 14.25 ± 7.59 s at 3 wkpi (*n* = 15), and 14.48 ± 10.28 s at 6 wkpi (*n* = 16), compared with 2.16 ± 2.02 s in sham controls (*n* = 27). There was no significant main effect of sex (*P* = 0.0748) and no sex-by-group interaction (*P* = 0.6345). Postoperative health was monitored using body weight trajectories from preoperative baseline through postoperative days 1–4 and at the time of electrophysiological assessment. While there was the expected effect of sex (*P* < 0.0001) associated with a difference in weight, there was no effect of injury or sex-by-injury interaction, suggesting that the mild CHI did not have lasting adverse effects on general health among the groups.

Figure 2 provides an overview of the experimental design (Fig. 2A), electrophysiological recording configuration (Fig. 2B), and representative field potential features (Fig. 2C) used for the analyses described below. In *ex vivo* slices, FV amplitude, fEPSP slope, and population spike threshold (see *Materials and methods*) were extracted from electrically-evoked field potentials in CA1 stratum radiatum and the molecular layer of the DG (Fig. 2C and D). To determine whether surgical manipulation alone affected synaptic outcomes, we compared electrophysiological measures between naïve and sham mice using a factorial model that included sex, surgical manipulation, and timepoint, stratified by hippocampal region. Representative field potentials acquired in both hippocampal subregions across timepoints and treatment groups are shown in Fig. 2E. To determine whether sham animals could be collapsed across timepoints, we performed factorial analyses including surgical condition (naive versus sham), sex, and timepoint. Across electrophysiological endpoints, there were no significant main effects of surgery and no surgery × timepoint interactions in either region, indicating that sham responses were stable across time and did not differ from naive controls. These results justified collapsing sham animals across timepoints for subsequent analyses. Two surgery × sex interactions were detected in CA1 for synaptic strength and synaptic efficiency (*P* = 0.036 and *P* = 0.037); however, no timepoint effects were observed in either region. Naive animals are shown for visual reference. Animal *n* values are now reported for each region-specific analysis, and differences in *n* between CA1 and DG reflect that not all animals yielded usable recordings in both regions.

**Figure 3:**
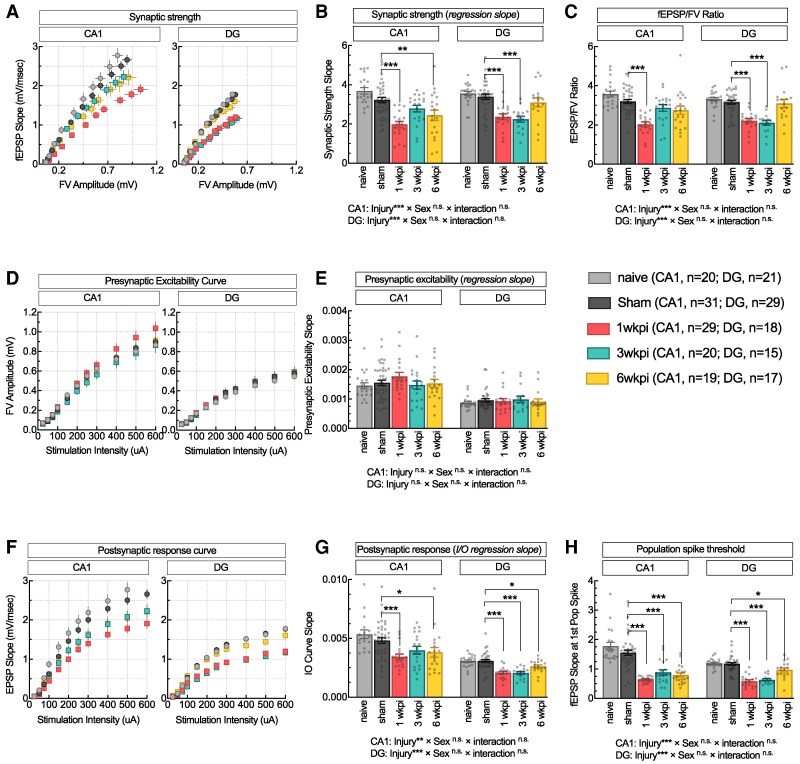
CHI alters hippocampal synaptic physiology in a region- and time-dependent manner. Ex vivo field excitatory postsynaptic potentials (fEPSPs) were recorded from hippocampal slices prepared from naïve, sham and CHI mice at 1, 3, or 6 weeks post-injury (wkpi). Recordings were obtained from CA1 following stimulation of Schaffer collateral fibres and from the dentate gyrus (DG) following stimulation of the perforant path. (**A**) Synaptic strength curve plotted as fEPSP slope versus fibre volley (FV) amplitude. (**B**) Synaptic strength quantified as the regression slope of panel A synaptic strength curves. (**C**) Synaptic efficiency measured as the fEPSP/FV ratio averaged across the three highest stimulation intensities. (**D**) Presynaptic excitability curve plotted as FV amplitude versus stimulus intensity. (**E**) Presynaptic excitability quantified as the regression slope of panel D presynaptic excitability curve. (**F**) Postsynaptic response input-output (I/O) curves plotting fEPSP slope as a function of stimulation intensity. (**G**) Postsynaptic responsiveness quantified as the regression slope of the panel F postsynaptic response curve. (**H**) Population spike threshold quantified as the fEPSP slope at which the first population spike was detected. Data are presented as mean ± SEM. CA1 and DG were analyzed separately. Statistical comparisons were performed using two-factor ANOVA with injury and sex as factors, followed by Dunnett’s post hoc tests where appropriate. Main effects of injury (exp_group) for each measure were as follows. Synaptic strength regression slope (**B**): CA1 *F*(3,90) = 9.47, *P* < 0.001; DG F(3,78) = 12.36, *P* < 0.001. Synaptic efficiency (**C**): CA1 *F*(3,90) = 9.85, *P* < 0.001; DG *F*(3,78) = 16.99, *P* < 0.001. Presynaptic excitability regression slope (**E**): no significant main effect of injury in either region (CA1 *F*(3,90) = 0.83, *P* = 0.479; DG *F*(3,78) = 0.19, *P* = 0.906). Postsynaptic responsiveness regression slope (**G**): CA1 *F*(3,90) = 5.13, *P* = 0.003; DG *F*(3,78) = 15.17, *P* < 0.001. Population spike threshold (**H**): CA1 *F*(3,90) = 25.00, *P* < 0.001; DG *F*(3,78) = 29.32, *P* < 0.001. Panel F shows the input–output curves; the corresponding statistics are reported for the regression slope in panel G. Each dot represents an individual animal (*n* values shown in legend); multiple slices per animal were averaged within each animal prior to statistical analysis. Animals examined per group: CHI 1 wkpi, 10 female/8 male; CHI 3 wkpi, 10 female/10 male; CHI 6 wkpi, 12 female/7 male; naïve, 8 female/13 male; sham, 25 female/17 male. Not all animals yielded usable recordings in both regions, so per-region *n* differs between CA1 and DG. Sham animals were collapsed across timepoints based on the absence of surgery and surgery × timepoint effects. * = *P* < 0.05, ** = *P* < 0.01, *** = *P* < 0.001.

After establishing that we could collapse the sham animals across timepoints, we then used a two-factor model including injury (sham versus 1, 3, and 6 wkpi) and sex and stratified the data by hippocampal region to evaluate the effects of CHI on synaptic physiology. In CA1, overall synaptic strength (Fig. 3A–C) was reduced following injury compared with sham controls. Synaptic strength (Fig. 3B), quantified by the regression slopes in Fig. 3A, provides a dynamic measure of how postsynaptic responses scale with increasing presynaptic input across stimulation intensities, capturing changes in input-output coupling across the full recruitment range. CHI reduced synaptic strength regression slope (*F*(3,90) = 9.47, *P* < 0.001, partial *η*^2^ = 0.240), with a reduction by 37% at 1 wkpi relative to sham (Δ = −1.196, *P* < 0.001), not significantly different at 3 wkpi (13% reduction; Δ = −0.405, *P* = 0.226), and reduced again at 6 wkpi (27% reduction; Δ = −0.849, *P* = 0.002). The synaptic efficiency, fEPSP/FV ratio (Fig. 3C) is a static measure calculated at the three highest stimulation intensities and reflects synaptic efficacy when presynaptic fibre recruitment is maximal. The CHI reduced synaptic efficiency in CA1 (*F*(3,90) = 9.85, *P* < 0.001, partial *η*^2^ = 0.247), driven by a reduction at 1 wkpi (35%; Δ = −1.112, *P* < 0.001), with no significant differences at 3 wkpi (9%; Δ = −0.290, *P* = 0.355) or 6 wkpi (13%; Δ = −0.398, *P* = 0.151). These measures show that CHI alters both dynamic synaptic coupling and maximal synaptic efficacy. There appeared to be little contribution of presynaptic excitability (Fig. 3D and E) as there was no main effect of injury. Maximal fEPSP amplitude was not significantly affected by injury, sex, or the sex × injury interaction in either CA1 or DG. In contrast to the largely intact presynaptic recruitment, postsynaptic responsiveness (Fig. 3F and G) was significantly affected by CHI. The postsynaptic input-output regression slope (Fig. 3G) showed a main effect of injury (*F*(3,90) = 5.13, *P* = 0.003, partial *η*^2^ = 0.146), with reductions relative to sham at 1 wkpi (29%, *P* = 0.002) and 6 wkpi (22%, *P* = 0.033), while the 3 wkpi comparison did not reach significance (19%, *P* = 0.065). These reductions in postsynaptic responsiveness persisted beyond the acute post-injury period in CA1.

Population spike threshold reflects the synaptic drive required to recruit synchronized firing and is commonly used as a field potential proxy for neuronal excitability.^64,65^ In CA1 (Fig. 3H), CHI resulted in a robust reduction in the population spike threshold relative to sham (*F*(3,90) = 25.00, *P* < 0.001, partial *η*^2^ = 0.455) across all timepoints (1 wkpi: 58%, Δ = −0.909, *P* < 0.001; 3 wkpi: 43%, Δ = −0.671, *P* < 0.001; 6 wkpi: 47%, Δ = −0.732, *P* < 0.001), consistent with enduring hyperexcitability.

The DG showed a similar profile of decreased synaptic strength, postsynaptic responsiveness, and increased neuronal excitability. In the DG synaptic strength regression slope (Fig. 3B) was decreased with CHI (*F*(3,78) = 12.36, *P* < 0.001, partial *η*^2^ = 0.322). Like the CA1 region, at 1 wkpi, the DG synaptic strength was reduced 31% (Δ = −1.050, *P* < 0.001). In contrast to the CA1 region, a deficit in synaptic strength regression slope was seen at 3 wkpi (34%; Δ = −1.142, *P* < 0.001), but not at 6 wkpi (7%; Δ = −0.246, *P* = 0.600), indicating a distinct temporal profile of recovery in DG compared with CA1. Synaptic efficiency in the DG (Fig. 3C) was similarly affected, with a robust main effect of injury (*F*(3,78) = 16.99, *P* < 0.001, partial *η*^2^ = 0.395) and significant reductions at 1 wkpi (31%; Δ = −0.964, *P* < 0.001) and 3 wkpi (33%; Δ = −1.048, *P* < 0.001), but not at 6 wkpi (1%; Δ = −0.029, *P* = 0.998). As in CA1, presynaptic excitability in DG (Fig. 3D and E) was not affected by injury. This finding indicates that axonal recruitment remains largely intact following CHI. However, postsynaptic responsiveness in DG (Fig. 3F and G) showed a larger and more persistent injury effect than in CA1, with the postsynaptic input-output regression slope (Fig. 3G) decreased following a CHI (*F*(3,78) = 15.17, *P* < 0.001, partial *η*^2^ = 0.368), with significant reductions at 1 wkpi (32%; Δ = −0.001, *P* < 0.001), 3 wkpi (33%; Δ = −0.001, *P* < 0.001), and 6 wkpi (15%; Δ = 0.000, *P* = 0.040). As in the CA1 region, there was an increased neuronal excitability in the DG, as shown by a lower population spike threshold with injury. This was seen at 1 wkpi (49%; Δ = −0.575, *P* < 0.001), 3 wkpi (45%; Δ = −0.531, *P* < 0.001), and 6 wkpi (16%; Δ = −0.189, *P* = 0.035). Across endpoints, sex effects were not significant, and sex-by-group interactions were not detected, indicating that the injury-related physiological changes were expressed similarly in males and females.

### Closed-head injury induces sex-dependent alterations in dendritic spine morphology and its relationship to synaptic physiology

**Figure 4:**
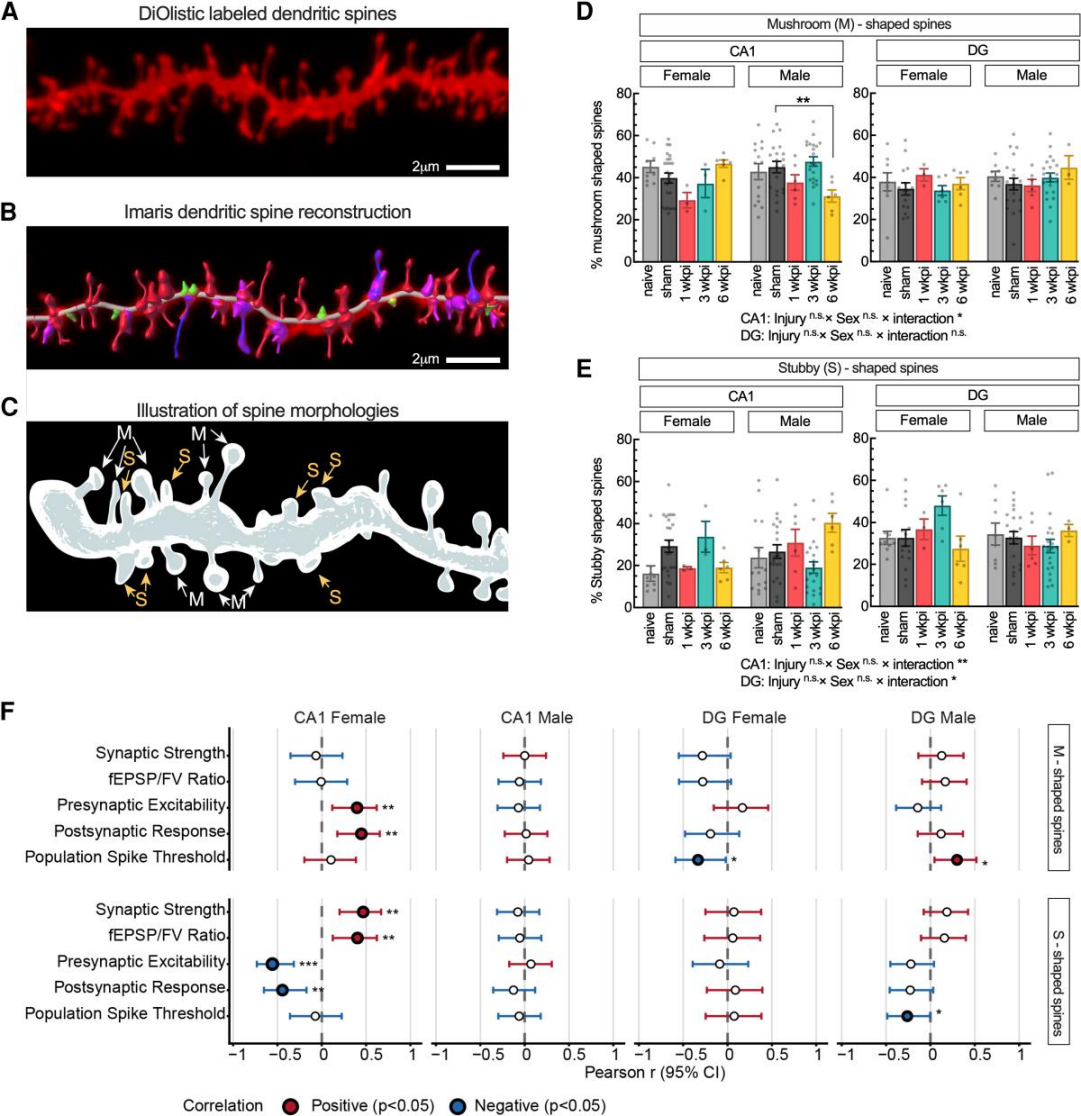
Sex-dependent effects of CHI on dendritic spine morphology and its relationship to synaptic physiology. (**A**) Representative DiOlistically labelled CA1 pyramidal neuron dendritic segments used for spine analysis. (**B**) Representative three-dimensional Imaris reconstruction, with mushroom spines shown in red, stubby spines in green, and ‘other’ spines in blue. (**C**) Schematic illustrating spine classification criteria used for analysis (mushroom, stubby, and other morphologies). Adapted with permission from Diamond DM, Campbell AM, Park CR, Woodson JC, Conrad CD, Bachstetter AD, Mervis RF. Influence of predator stress on the consolidation versus retrieval of long-term spatial memory and hippocampal spinogenesis. Hippocampus. 2006;16(7):571–576. Copyright © 2006 Wiley-Liss, Inc. This panel is intended to illustrate the classification scheme. Quantification of mushroom-shaped (**D**) and stubby-shaped (**E**) spine proportions in CA1 following sham or CHI, stratified by sex. (**F**) Correlation analyses between dendritic spine morphology and electrophysiological measures obtained from the same animals in CA1 and DG. Data are presented as mean ± SEM. Statistical comparisons in (**D**) and (**E**) used two-way ANOVA with injury and sex as factors. In CA1, there was a significant sex × injury interaction for both mushroom-shaped spines (D; *F*(3,81) = 3.86, *P* = 0.012) and stubby-shaped spines (E; *F*(3,81) = 4.37, *P* = 0.007). Sex-stratified analysis showed a significant effect of injury in males for mushroom-shaped spines (*F*(3,49) = 4.55, *P* = 0.007) and stubby-shaped spines (*F*(3,49) = 4.31, *P* = 0.009), with no significant injury effects in females. CA1 segment counts: naïve *n* = 9F/14M; sham *n* = 24F/21M; 1 wkpi *n* = 3F/6M; 3 wkpi *n* = 3F/21M; 6 wkpi *n* = 6F/6M. DG segment counts: naïve *n* = 9F/9M; sham *n* = 15F/18M; 1 wkpi *n* = 3F/6M; 3 wkpi *n* = 6F/21M; 6 wkpi *n* = 6F/3M. In (**F**), each dot represents the Pearson correlation coefficient with 95% confidence interval between the proportion of mushroom- or stubby-shaped spines and an electrophysiology measure within the matched animal subset. Filled circles indicate *P* < 0.05. *P < 0.05, **P < 0.01, ***P < 0.001.

To determine whether alterations in dendritic spine morphology accompanied the synaptic dysfunction observed following CHI, we used DiOlistic labelling and three-dimensional Imaris reconstruction (Fig. 4A–C). Total spine density was not significantly affected by injury group in either CA1 (*P* = 0.5962) or DG (*P* = 0.9649), and there was no significant effect of sex or sex × injury interaction in either region. Spines were classified as mushroom, stubby, or other. The percentage of ‘other’ spines was not significantly affected by injury, sex, or the sex × injury interaction in either CA1 or DG. Percent mushroom and percent stubby were calculated relative to the total detected spine population, with other spines retained in the denominator but not analyzed as an independent category. Using a two-way ANOVA with injury and sex as main factors, stratified by hippocampal region, we found sex × injury interactions in the CA1 region for both mushroom-shaped spines (*F*(3,81) = 3.86, *P* = 0.012, partial *η*^2^ = 0.12; Fig. 4D) and stubby-shaped spines (*F*(3,81) = 4.37, *P* = 0.007, partial *η*^2^ = 0.13; Fig. 4E), indicating that injury-related changes in spine morphology differed between males and females. Stratification by sex revealed that CHI significantly altered the percentage of mushroom-shaped spines (*F*(3,49) = 4.55, *P* = 0.007, partial *η*^2^ = 0.218) and stubby-shaped spines (*F*(3,49) = 4.31, *P* = 0.009, partial *η*^2^ = 0.209) in males, but not females, with post hoc comparisons demonstrating a reduction in mushroom-shaped spines at 6 weeks post-injury relative to sham controls (Δ = −13.8%, 95% CI [−25.8, −1.8], *P* = 0.020; Fig. 4D) and a corresponding increase in stubby-shaped spines (Fig. 4E).

Because dendritic spine morphology and synaptic physiology were assessed in the same animals, we next examined the relationship between structural and physiological endpoints using Pearson correlation analyses (Fig. 4F). In CA1 of female mice, mushroom-shaped spines were positively correlated with synaptic strength and postsynaptic responsiveness, whereas stubby-shaped spines showed inverse relationships with these same measures (*P* < 0.05). Presynaptic excitability was correlated with both mushroom- and stubby-shaped spine proportions. These two spine classes were inversely correlated with one another. In contrast, no significant correlations were detected in CA1 males between spine morphology and electrophysiological endpoints despite the presence of injury-related changes in spine class proportions at later timepoints. In the DG, population spike threshold was significantly correlated with spine morphology in both sexes (*P* < 0.05); however, the direction of the association between mushroom-shaped spines and population spike threshold differed between females and males (Fig. 4F), indicating sex-specific relationships between dendritic structure and neuronal excitability in this region. While synaptic electrophysiological parameters did not show sex or sex-by-injury interaction effects, dendritic spine morphology revealed sex-dependent injury effects in CA1.

### Histopathological assessment of glial markers in hippocampal subregions

**Figure 5:**
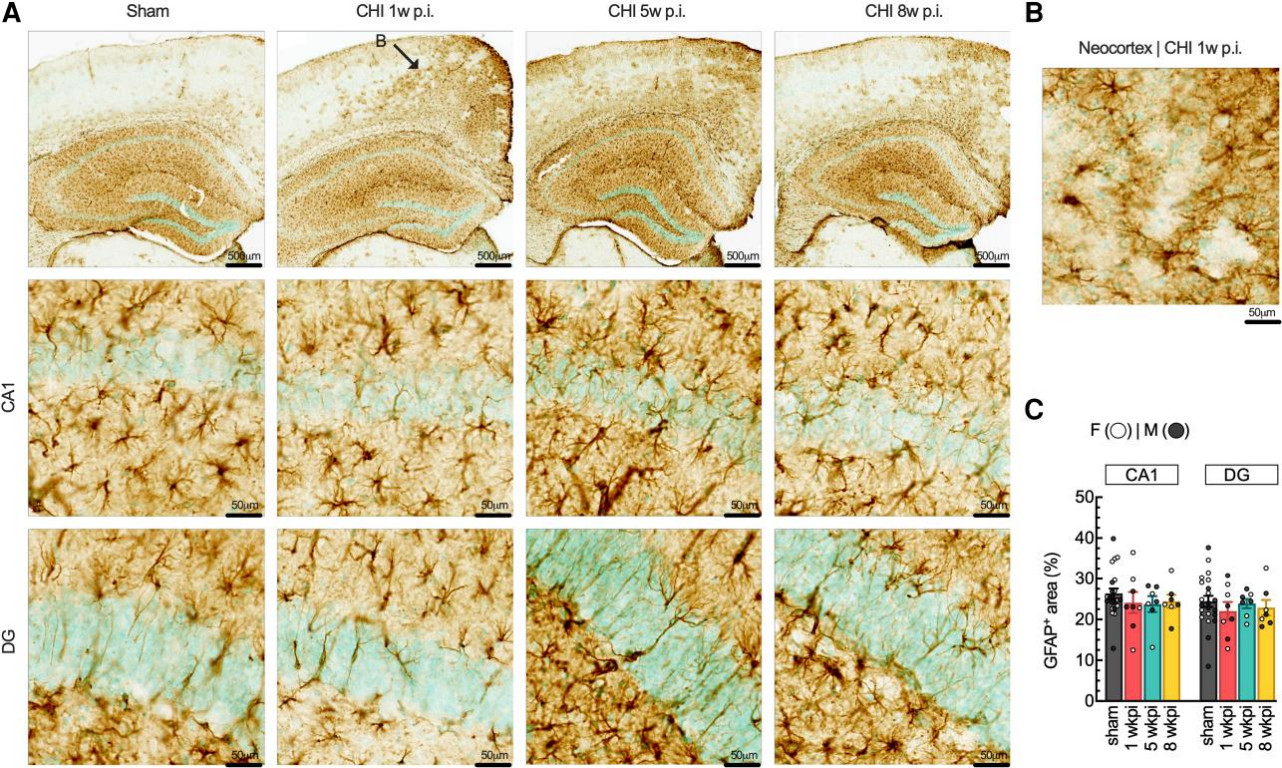
Astrocyte reactivity following CHI assessed by GFAP immunohistochemistry. (**A–B**) Representative GFAP immunostaining in neocortex and hippocampus from Sham and CHI mice at 1, 5, and 8 weeks post-injury (wkpi). Neocortical GFAP expression was elevated at 1 wkpi, consistent with astrocyte activation near the impact site. Within hippocampus, GFAP labelling in CA1 and DG appeared uniform across groups, with no qualitative evidence of hypertrophy. **(C)** Quantification of GFAP^+^ area in CA1 and DG showed no significant effects of region, injury, sex, or timepoint (Main effects and interactions were all non-significant: region *F*(1,72) = 0.89, *P* = 0.348; injury *F*(3,72) = 0.83, *P* = 0.481; sex *F*(1,72) = 0.34, *P* = 0.564; sex × injury *F*(3,72) = 1.38, *P* = 0.256). Percent area fraction was analyzed using mixed-effects ANOVA with animal as a random intercept and injury, timepoint, region, and sex as fixed effects, followed by Dunnett’s or Tukey’s corrected post hoc comparisons where applicable. Each data point represents the percent area fraction from an individual mouse for the indicated hippocampal region. Shams were collected at each time point and collapsed into a single control group for analysis and presentation. Data from females and males are shown together for visualization, but sex was included as a factor in the statistical model. Sex-specific post hoc comparisons are indicated where applicable. Sample sizes were: sham, 1 wkpi *n* = 6F/8M; sham, 5 wkpi *n* = 8F/8M; sham, 8 wkpi *n* = 8F/6M; CHI, 1 wkpi *n* = 8F/8M; CHI, 5 wkpi *n* = 6F/8M; CHI, 8 wkpi *n* = 6F/8M. Data represent mean ± SEM.

**Figure 6:**
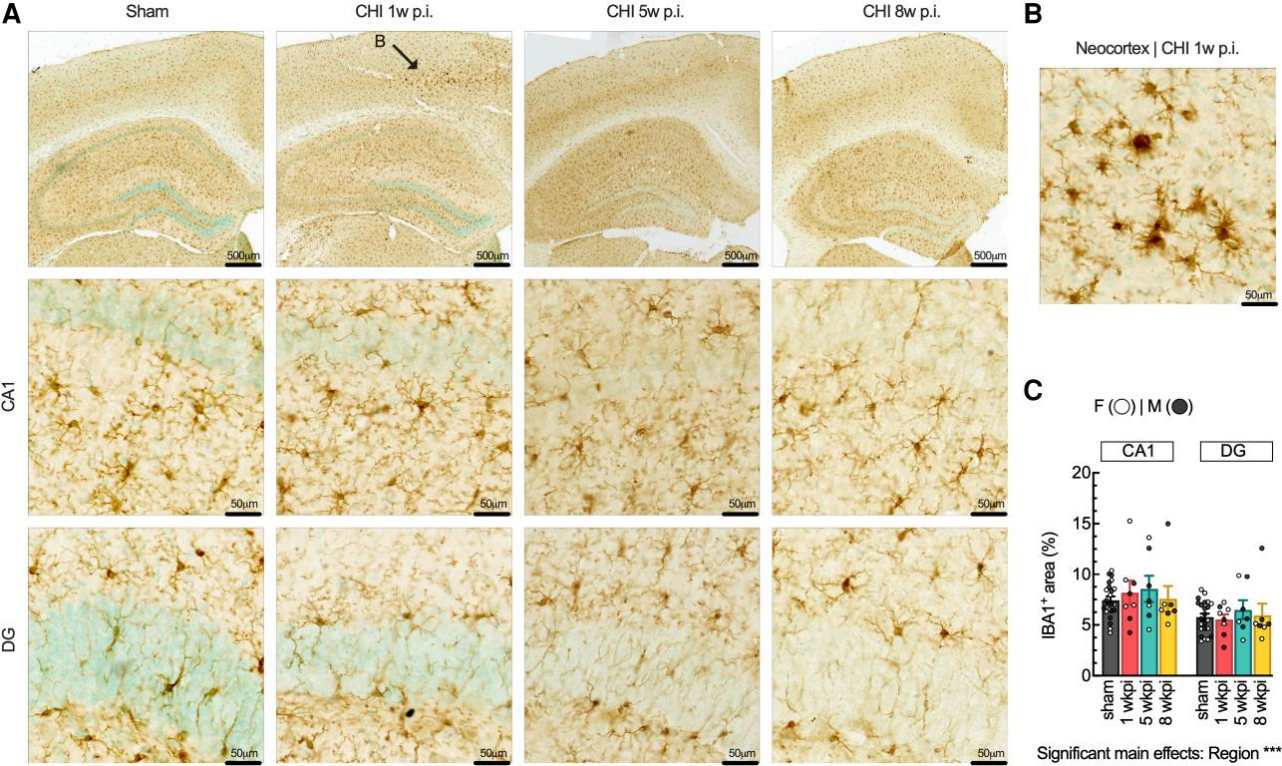
Microglial response after CHI assessed by IBA1 immunohistochemistry. (**A–B**) Representative IBA1 staining shows robust cortical microglial activation at 1 week post-injury (wkpi), which resolved by 8 wkpi. (**C**) In CA1 and DG, IBA1^+^ area showed no effects of injury or sex; the only significant factor was a main effect of region (*F*(1,74) = 13.04, *P* = 0.0006, partial *η*^2^ = 0.150), with CA1 exhibiting greater IBA1 coverage than DG. Non-significant effects: injury *F*(3,74) = 0.56, *P* = 0.640; sex *F*(1,74) = 0.22, *P* = 0.641. Percent area fraction was analyzed using mixed-effects ANOVA with animal as a random intercept and injury, timepoint, region, and sex as fixed effects, followed by Dunnett’s or Tukey’s corrected post hoc comparisons where applicable. Each data point represents the percent area fraction from an individual mouse for the indicated hippocampal region. Shams were collected at each timepoint and collapsed for statistical analysis. Data from females and males are shown together for visualization, but sex was included as a factor in the statistical model. Sex-specific post hoc comparisons are indicated where applicable. Sample sizes were: sham, 1 wkpi *n* = 6F/8M; sham, 5 wkpi *n* = 8F/8M; sham, 8 wkpi *n* = 8F/8M; CHI, 1 wkpi *n* = 8F/8M; CHI, 5 wkpi *n* = 6F/8M; CHI, 8 wkpi *n* = 6F/8M. Data represent mean ± SEM.

**Figure 7:**
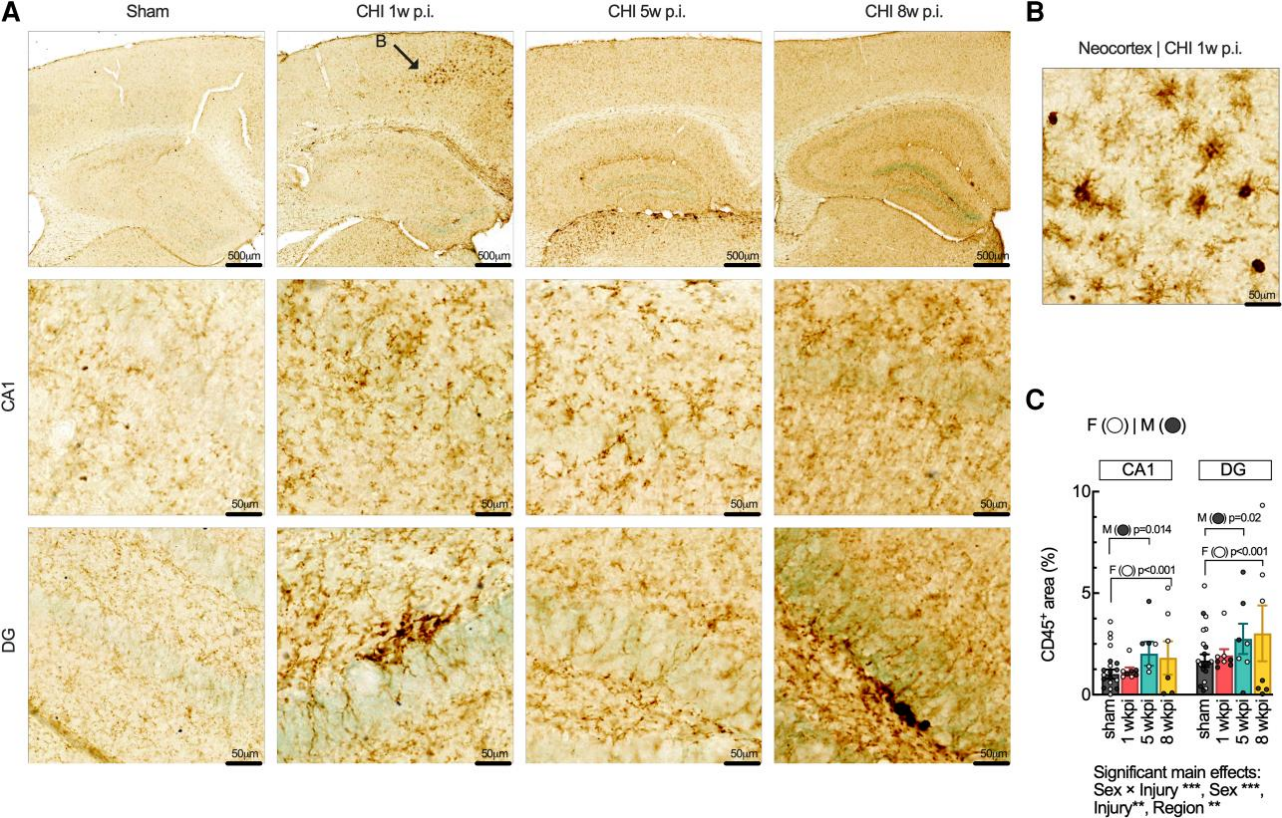
CD45 immunoreactivity reveals delayed and sex-dependent changes in hippocampus after CHI. (**A–B**) CD45 staining showed strong neocortical activation at 1 week post-injury (wkpi). (**C**) In CA1 and DG, CD45^+^ area displayed significant main effects of sex (*F*(1,74) = 19.02, *P* < 0.0001, partial *η*^2^ = 0.205), injury (*F*(3,74) = 5.35, *P* = 0.0022, partial *η*^2^ = 0.178), and region (*F*(1,74) = 7.98, *P* = 0.0061, partial *η*^2^ = 0.097), as well as a significant sex × injury interaction (*F*(3,74) = 15.20, *P* < 0.0001, partial *η*^2^ = 0.381). Post hoc tests showed increased CD45^+^ area at 5 wkpi in males and at 8 wkpi in females. Percent area fraction was analyzed using mixed-effects ANOVA with animal as a random intercept and injury, timepoint, region, and sex as fixed effects, followed by Dunnett’s or Tukey’s corrected post hoc comparisons where applicable. Each data point represents the percent area fraction from an individual mouse for the indicated hippocampal region. Shams were collected at each time point and collapsed into a single group for presentation. Data from females and males are shown together for visualization, but sex was included as a factor in the statistical model. Sex-specific post hoc comparisons are indicated where applicable. Sample sizes were: sham, 1 wkpi *n* = 6F/8M; sham, 5 wkpi *n* = 8F/8M; sham, 8 wkpi *n* = 8F/8M; CHI, 1 wkpi *n* = 8F/8M; CHI, 5 wkpi *n* = 6F/8M; CHI, 8 wkpi *n* = 6F/8M. Data represent mean ± SEM.

Having observed region-specific alterations in electrophysiology and no change in total dendritic spine density following CHI, we next evaluated GFAP, IBA1, and CD45 immunoreactivity in CA1 and DG using archived tissue from an independent histology cohort collected at 1, 5, and 8 weeks post-injury. Prior work from our group demonstrated robust and lasting astrocyte and microglial activation in the neocortex near the impact site, but comparable analyses in hippocampal subregions have not been performed.^63^ Consistent with previous observations, GFAP staining was markedly elevated in the neocortex at 1 wkpi (Fig. 5A and B), but within the hippocampus GFAP expression was uniformly high across all groups, including shams, and astrocyte morphology in CA1 and DG showed no qualitative or quantitative differences. Digital quantification confirmed no significant effects of region, injury, sex, or timepoint on GFAP^+^ area (Fig. 5C). Similarly, IBA1 revealed dense microglial activation in the cortex at 1 wkpi that resolved by 8 wkpi (Fig. 6A and B), but hippocampal IBA1 staining showed no injury- or sex-dependent differences. The only significant effect was region, with CA1 exhibiting higher IBA1^+^ area than DG (*F*(1,74) = 13.04, *P* = 0.0006, partial *η*^2^ = 0.150) (Fig. 6C). In contrast, CD45, a marker labelling reactive microglia and other leukocytes, showed pronounced cortical activation at 1 wkpi (Fig. 7A and B) and, uniquely among the markers assessed, demonstrated persistent and injury-related changes within the hippocampus. Quantification revealed significant main effects of sex (*F*(1,74) = 19.02, *P* < 0.0001, partial *η*^2^ = 0.205), injury (*F*(3,74) = 5.35, *P* = 0.0022, partial *η*^2^ = 0.178) and region (*F*(1,74) = 7.98, *P* = 0.0061, partial *η*^2^ = 0.097), as well as a significant sex × injury interaction (*F*(3,74) = 15.2, *P* < 0.0001, partial *η*^2^ = 0.381) (Fig. 7C). Stratified analyses revealed that males exhibited increased CD45^+^ area in both CA1 and DG at 5 wkpi, whereas females showed a delayed increase emerging at 8 wkpi; males displayed partial resolution by this later timepoint. These findings indicate that while astrocyte and microglial morphology remain largely unchanged in CA1 and DG, CD45-positive cell immunoreactivity demonstrates a delayed and sex-dependent increase in the chronic period. Because histology and electrophysiology were performed in independent cohorts, these findings should be interpreted as complementary assessments of delayed hippocampal pathology rather than as direct mechanistic correlates of the physiological changes.

## Discussion

This study tested whether mild diffuse CHI produces lasting hippocampal dysfunction concurrently with early transcriptional, synaptic, structural, and neuroimmune correlates. We found that CHI induced rapid transcriptional changes within the first 9–24 h, including immediate early genes, excitability regulators, and synaptic factors, but most of these changes had largely resolved by 72 h. Despite this transient molecular response, both CA1 and DG showed aberrant synaptic function that lasted through 6 weeks post-injury. Notably, reduced synaptic strength occurred alongside chronic hyperexcitability, with population spikes emerging at lower thresholds and requiring less synaptic drive to elicit synchronized firing. This combination of weakened synaptic transmission but heightened neuronal excitability has direct relevance to post-traumatic seizure risk. These circuit-level changes were accompanied by shifts toward more immature spine morphologies. Notably, this dysfunction occurred despite a lack of overt hippocampal pathology in this injury model, as damage was largely limited to the cortex near the impact site. Hippocampal GFAP and IBA1 were largely unchanged, and only CD45 showed delayed increases in hippocampal subregions, with sex-dependent timing. Together, these data suggest that mild CHI produces chronic hippocampal circuit dysfunction, including a hyperexcitable state that may begin with early transcriptional changes, but can persist long after those molecular responses have normalized.

In this CHI model, prior work has demonstrated persistent behavioural impairments, including deficits in hippocampal-dependent learning and memory tasks.^62,63,66-69^ The present findings provide circuit-level context for these deficits, showing that reductions in synaptic strength and increased neuronal excitability in CA1 and DG are present during overlapping post-injury intervals. Prior work in diffuse and focal injury models reports reduced long-term potentiation, altered excitatory drive, and disrupted oscillatory dynamics after mild injury.^14,18,24,43,45,46^ We extend these findings by characterizing CA1 and DG input-output relationships across subacute and chronic intervals within the same experimental design. In CA1, synaptic strength and postsynaptic responses were reduced at 1 week and showed signs of recovery at 3 weeks. However, deficits re-emerged at 6 weeks, suggesting that early recovery may not reflect lasting circuit restoration. In DG, synaptic strength was reduced at 1 and 3 weeks but recovered by 6 weeks. Prior work showing disrupted AMPA receptor trafficking and phosphorylation after mild TBI provides a plausible context for these reductions in synaptic strength.^17,30,44^ This region-specific vulnerability aligns with evidence that CA1 pyramidal neurons show heightened susceptibility to metabolic stress and excitotoxic signalling, whereas DG granule cells exhibit circuit features, including robust inhibitory gating and ongoing adult neurogenesis, that may support resilience and recovery.^24,29,33,70^ The temporal profile of DG recovery observed here parallels reports of adult-born granule cell integration following TBI, which may restore synaptic throughput over time.

Presynaptic recruitment (FV amplitude) remained intact across stimulation intensities, supporting the inference that reduced synaptic strength slopes and reduced fEPSP/FV ratios reflect altered postsynaptic mechanism downstream of recruited presynaptic fibres. This differs from some fluid percussion and controlled cortical impact datasets reporting reduced presynaptic excitability.^14,24^ The present data extend these observations to a diffuse CHI model, demonstrating that postsynaptic impairments occur even in the absence of overt focal damage. Persistent reductions in fEPSP slope at higher stimulation intensities in CA1 suggest impaired synaptic summation or receptor recruitment, consistent with altered glutamate receptor composition or reduced postsynaptic spine efficiency.^39,44,71^

Although total dendritic spine density remained unchanged across conditions, DG neurons exhibited significantly higher stubby spine density relative to CA1, independent of injury status. Stubby spines are typically associated with developing or transient synaptic connections, and their predominance in DG may reflect ongoing structural plasticity or neurogenesis rather than injury-induced degeneration.^38,40,42,72^ The lack of change in mushroom-type spines, usually indicative of stable, mature synapses, suggests that physiological deficits observed here are not accompanied by global synaptic loss but rather by subtle modifications in receptor density or local signalling. This interpretation is consistent with previous work showing that hippocampal dendritic spines undergo dynamic remodelling rather than uniform loss after mild or moderate TBI.^38,39,42^ The higher prevalence of stubby spines in DG may therefore represent a structural correlate of recovery, consistent with the restoration of synaptic strength observed electrophysiologically at 6 weeks post-injury.

Our data also shows that CHI produces persistent (1, 3, and 6 weeks post-injury), regionally patterned changes in hippocampal hyperexcitability. Because FV recruitment was preserved, these population spike phenotypes are unlikely to reflect failure of presynaptic activation and instead support circuit-level mechanisms involving altered postsynaptic mechanisms and/or excitation-inhibition balance. We observed transient transcriptional downregulation of *Kcnt2* and *Kcnc3*. Loss of *Kcnt2* (Slick) removes a critical sodium-activated ‘safety brake’ against repetitive firing, while reduced expression of *Kcnc3* (Kv3.3) likely impairs the fast repolarization required for effective network inhibition. These molecular changes may contribute to destabilization of circuit function, favouring a hyperexcitable state despite concurrent reductions in synaptic strength, which would need to be experimentally tested. Notably, the combination of depressed synaptic strength alongside heightened network excitability is a recurring phenotype across multiple neurologic disease models, including experimental TBI and several astrocyte-/Aβ-linked neurodegenerative and cerebrovascular paradigms, suggesting shared circuit-level failure modes that decouple synaptic drive from spike output.^14,47,73-77^ However, prior work also links post-TBI hippocampal hyperexcitability to impaired inhibitory control, altered intrinsic membrane properties, and disrupted ionic homeostasis.^48,49,78,79^ Candidate mechanisms include reduced hyperpolarization-activated currents, potassium channel dysfunction, and dysregulated calcium handling, each of which can increase firing probability and destabilize network timing.^48,49,78,79^ In CA1 specifically, several studies implicate altered NMDA receptor-dependent calcium influx, and related calcium-dependent injury cascades as contributors to chronic excitability shifts after injury.^80-82^ Glutamatergic dysfunction can be driven by glial D-serine release, which hyperactivates perisynaptic GluN2B-containing NMDARs to trigger complement-mediated synaptic pruning in the absence of cell death.^83^

In DG, prolonged hyperexcitability has been attributed to reduced GABAergic inhibition, impaired KCC2-mediated chloride extrusion, and network remodelling that weakens feed-forward inhibition, with implications for seizure susceptibility in some models.^14,24,26,79,84^ While the precise hierarchy of these molecular and physiological changes remains to be defined, these findings indicate that the balance of excitation and inhibition is persistently disrupted following a mild closed head injury.

The increased excitatory potential and reduced synaptic strength observed in CA1 and DG were not accompanied by changes in hippocampal gliosis markers such as GFAP or IBA1, despite robust cortical gliosis persisting at chronic time points proximal to the injury site. CD45-positive cell immunoreactivity, however, showed a delayed and sex-dependent increase within hippocampal subregions. Together, these findings indicate that hippocampal circuit dysfunction after mild diffuse TBI can persist independently of overt regional gliosis, underscoring a dissociation between synaptic pathology and overt GFAP/IBA1-defined gliosis while not excluding contributions from specific cellular phenotypes or pathological changes not captured by these measures.^85^ Although GFAP and IBA1 did not reveal overt hippocampal gliosis, this does not exclude other forms of pathology within hippocampal circuits. The present analyses were not designed to assess neuronal loss or cell type-specific vulnerability. In particular, loss or dysfunction of inhibitory interneurons, including hilar interneurons in the dentate gyrus, is a well-established mechanism contributing to hippocampal hyperexcitability after brain injury.^36,47,86,87^ While our electrophysiological data demonstrate preserved presynaptic recruitment alongside reduced postsynaptic responses and increased excitability, these measures do not resolve the relative contributions of inhibitory versus excitatory neuronal populations. Notably, preserved fibre volley recruitment argues against gross loss of afferent input, suggesting that the observed changes reflect circuit-level dysfunction rather than widespread structural degeneration. Thus, alterations in inhibitory circuitry remain a plausible contributor to the observed hyperexcitable phenotype and warrant direct investigation in future studies.

Several considerations should be noted. Bulk RNA sequencing does not resolve transcriptional changes within discrete hippocampal subregions or attribute them to specific cell types. Our electrophysiological analyses focused on overall synaptic function rather than receptor-specific mechanisms, and confirmatory patch-clamp or pharmacological experiments would be needed to identify precise postsynaptic contributions. Similarly, spine analyses were limited to morphological metrics, and molecular assays for synaptic proteins could clarify whether morphological stability conceals receptor-level changes.

## Data Availability

Bulk RNA-sequencing raw and processed data have been deposited at the Gene Expression Omnibus (GEO) under accession GSE326452. Electrophysiology, dendritic spine morphology, and immunohistochemistry datasets are publicly available at the Open Data Commons for Traumatic Brain Injury: electrophysiology (ODC-TBI:1593, https://doi.org/10.34945/F5R886), dendritic spine morphology (ODC-TBI:1594, https://doi.org/10.34945/F5MK69), and immunohistochemistry (ODC-TBI:1595, https://doi.org/10.34945/F5GS30). RNA-sequencing analysis used the nf-core/rnaseq pipeline (v3.21.0), DESeq2 (v1.46.0), clusterProfiler (v4.14.6), and ReactomePA (v1.50.0) with parameters reported in Methods. Figure visualizations were generated using standard plotting functions from these packages and ggplot2 with parameters reported in Methods. Additional analytical details are available from the corresponding author upon reasonable request. Tissue samples are not available for future use.
